# Supplementary figures and images for: Anopheles mosquito surveillance in Madagascar reveals multiple blood feeding behavior and Plasmodium infection
Source: PLoS Negl Trop Dis. 2019 Jul 5;13(7):e0007176. doi: 10.1371/journal.pntd.0007176 (PMC6663035; doi:10.1371/journal.pntd.0007176)

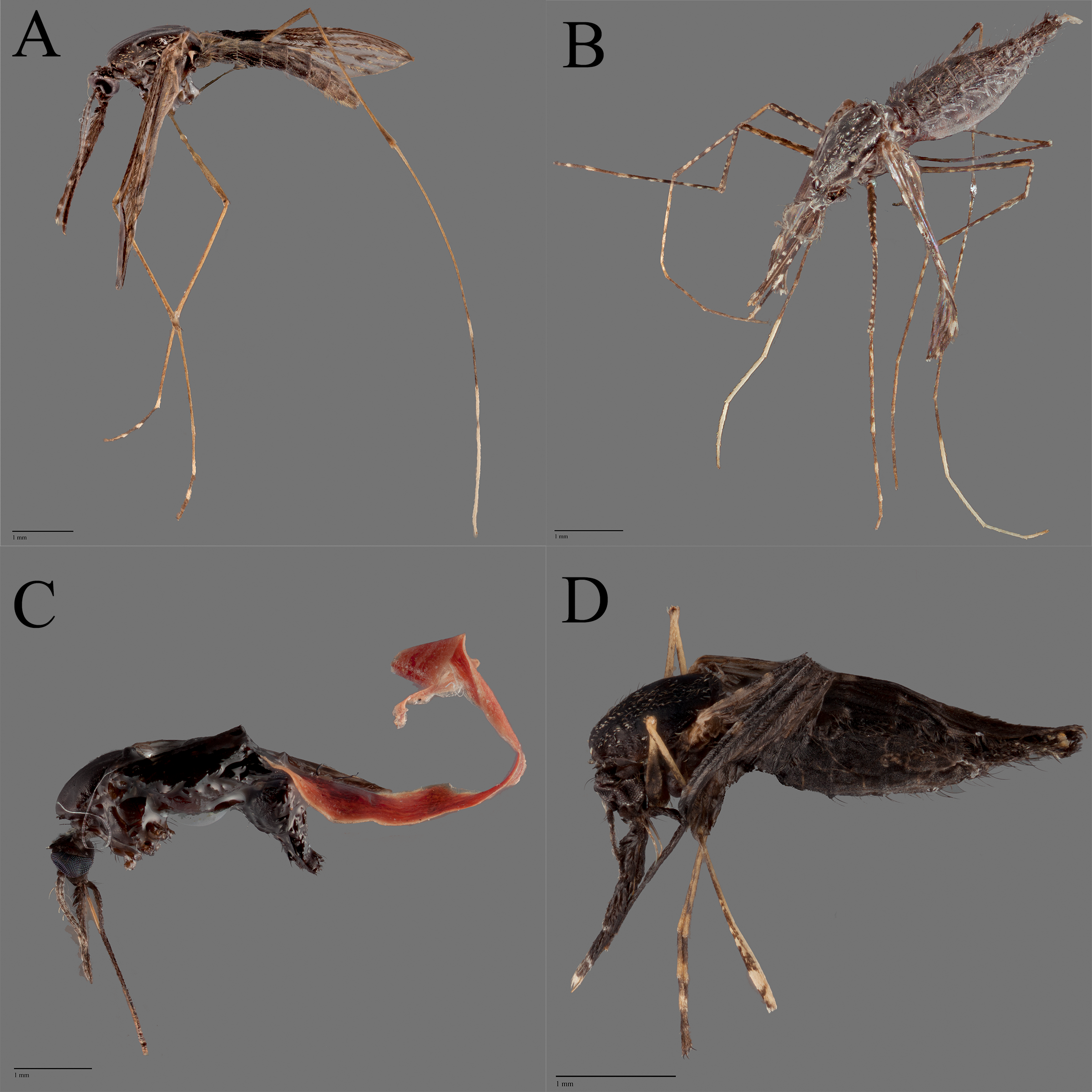

Supplement: S1 Fig — (A) Anopheles coustani (APY1071), (B) Anopheles maculipalpis (APY684), (C) Anopheles rufipes (APY1016), (D) Anopheles squamosus (APY702). (TIF) [file pntd.0007176.s001.tif]

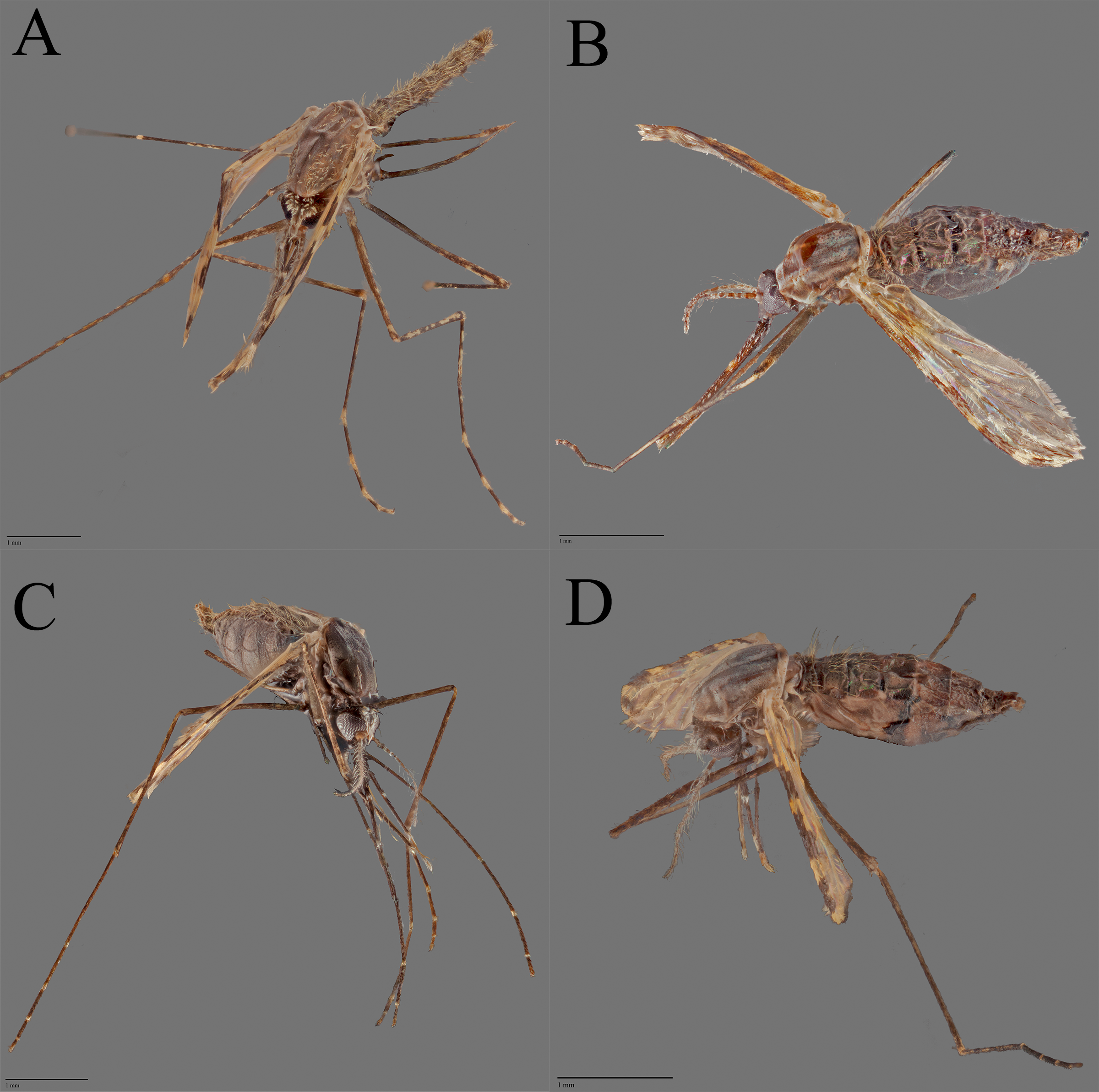

Supplement: S2 Fig — (A) Anopheles arabiensis (APY689), (B) Anopheles funestus (APY237), (C) Anopheles mascarensis (APY437), (D) Anopheles mascarensis (APY733). (TIF) [file pntd.0007176.s002.tif]

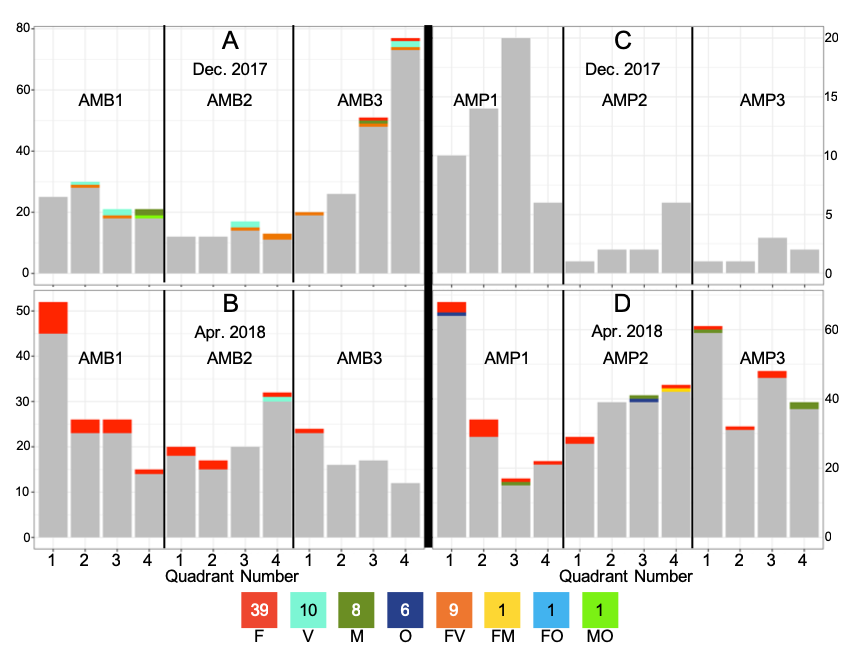

Supplement: S3 Fig — F = P. falciparum, V = P. vivax, M = P. malariae, O = P. ovale. Letter combinations indicate the presence of two or three species in the bloodmeal. Quadrant numbers refer to the direction of their orientation: 1-Northwest, 2-Northeast, 3-Southwest, 4-Southeast. (A) Ambolodina December 2017, (B) Ambolodina April 2018, (C) Amparihy December 2017, (D) Amparihy April 2018. Numerical version available in S6 Table. (PNG) [file pntd.0007176.s003.png]
