# Supplementary material for: Anopheles mosquito surveillance in Madagascar reveals multiple blood feeding behavior and Plasmodium infection
Source: PLoS Negl Trop Dis. 2019 Jul 5;13(7):e0007176. doi: 10.1371/journal.pntd.0007176 (PMC6663035; doi:10.1371/journal.pntd.0007176)
Supplement: S1 Table — (DOCX) [file pntd.0007176.s004.docx]

**Trap Coordinates**

QUEST (Quadrant Enabled Barrier Screen) (Fig 2A-D)

Amparihy Trap 1: S 19°23.112’, E 046°08.186’; 763m altitude

Amparihy Trap 2: S 19°23.089’, E 046°08.176’; 775m altitude

Amparihy Trap 3: S 19°23.059’, E 046°08.220’ ; 779m altitude

Ambolodina Trap 1: S 19°22.460’, E 046°07.459’; 808m altitude

Ambolodina Trap 2: S 19°22.457’, E 046°07.405’; 803m altitude

Ambolodina Trap 3: S 19°22.458’, E 046°07.356’; 802m altitude

Standard Barrier Screen (Fig 2E-F)

Ambolodina Trap 4: S 19°22.466’, E 046°07.439’; 809m altitude
