# Supplementary material for: Anopheles mosquito surveillance in Madagascar reveals multiple blood feeding behavior and Plasmodium infection
Source: PLoS Negl Trop Dis. 2019 Jul 5;13(7):e0007176. doi: 10.1371/journal.pntd.0007176 (PMC6663035; doi:10.1371/journal.pntd.0007176)
Supplement: S2 Table — (DOCX) [file pntd.0007176.s005.docx]

**Supplemental Table 2.) BLOODART Ligase Detection Reaction probes for the detection of mammalian host and mosquito species.**

| **Probe Name** | **Probe Sequence** | **FlexMap Microsphere** |
| --- | --- | --- |
| Dog | caaatacataatcttacattcactACATCACTTATTGATCCAATAATTT | 13 |
| Cat 2.0 | catcttcatatcaattctcttattTAGACTAACCAGTCGAAAGTACTAC | 35 |
| Human 2.0 | tctctttaaacacattcaacaataAGAACCCAACCTCCGAGCAGTACAT | 47 |
| Goat 2.0 | attaaacaactcttaactacacaaCTAGACTTACAAGTCAAATCAAATT | 36 |
| Cow 3.0 | atactttacaaacaaataacacacACTAGACCCACAAGTCAAATCACT | 19 |
| Pig 2.0 | tacttctttactacaatttacaacGACAAACCAGTCAAAATAACCATAC | 15 |
| Lemur | attcaatactatctaacacttactCTACAATCAATTGACCCAAACTAGT | 38 |
| Mouse | cactacacatttatcataacaaatCATATCTTATTGACCCARATATATT | 42 |
| LDR Mammal Common 1.2 | 5'-P-TTGAYCAAYGGAMCAAGTTACCCTAGGGAT-3’-B | - |
| LDR Mammal Common 2.0 | 5'-P- ATCRCTTATTGATCCAAAAA-3’-B | - |
| LDR Mammal Common 3.0 | 5'-P-GCTAAGACTTCACCAGTCAAAGCGA-3’-B | - |
| LDR Mammal Common 4.2 | 5'-P-CTATCGCTCATTGATCCAAAAACTTGATCA-3’-B | - |
| gambiae 2.0 | ctttcttaatacattacaacatacACGCACTGGGCGGTCGCTGTGCATA | 25 |
| arabiensis 2.0 | caaacaaacattcaaatatcaatcACGCACTGGGCGGCCGCTGTGCATG | 22 |
| mascarensis | tacattcaacactcttaaatcaaaGCTACTTTTTACCATTTCACCCAAC | 26 |
| rufipes | taacttacacttaactatcatcttTCGGGTCCGTGCCAAGCGCTTATGA | 27 |
| maculipalpis | cacttaattcattctaaatctatcCTATACACCGGCCGCCATAACAACA | 28 |
| funestus | tactacttctataactcacttaaaTTGTGAAACATGGGGAAATTCAATC | 29 |
| coustani | acttatttcttcactactatatcaGCAAGCCAGATGTGACGATAACAA | 34 |
| squamosus | tacacaatattcatcataactaacCAGGTTTGAGAATAGGGTTTAGATC | 45 |
| LDR MOSQ Common 1.2 | 5'-P- ATGACGTGCTTGGTCCCCGTCTGCGGGTCC -3’-B | - |
| LDR MOSQ Common 2 | 5'-P- GTAGGCCTCAAGTGATGTGTGACAACCCCCTG -3’-B | - |
| LDR MOSQ Common 3 | 5'-P- GAAAACCTCTTTGATGTCCAAGATTTCGTTGACCG -3’-B | - |
| LDR MOSQ Common 4.2 | 5'-P- CTATCGCTCATTGATCCAAAAACTTGATCA -3’-B | - |
| LDR MOSQ Common 5 | 5'-P- GTAGGCACTCAAGGATGTGTGCATCGGTCG -3’-B | - |

The species-specific BLOODART LDR primers correspond to a numbered FlexMap microsphere. The lowercase portion of these primers indicate the TAG sequence added to the 5’ end of the uppercase species-specific sequence. Lemur primers were built to amplify a diversity of lemur sequences, but were only tested on the ringtail lemur (*Lemur catta*), Coquerel’s sifaka (*Propithecus coquereli*).
